# Electronic Cigarette Use and Sleep Quality Among Young Adults in Jazan, Saudi Arabia: A Community-Based Cross-Sectional Study

**DOI:** 10.3390/healthcare14142068

**Published:** 2026-07-10

**Authors:** Majed A. Alotayfi, Sarah Salih, Abdulilah Alqarny, Ahmed M. Hashim, Atheer Homadi, Khalid Humedi, Reema Nharri, Ghada A. Ageeli

**Affiliations:** 1 Ministry of Health, Jazan 82816, Saudi Arabia; asalqarny@moh.gov.sa; 2 Department of Family & Community Medicine, Jazan University, Jazan 82816, Saudi Arabia; ssalih@jazanu.edu.sa; 3 College of Medicine, Jazan University, Jazan 82816, Saudi Arabia; 202300787@stu.jazanu.edu.sa (A.M.H.); 202203471@stu.jazanu.edu.sa (A.H.); 202301838@stu.jazanu.edu.sa (K.H.); 202203504@stu.jazanu.edu.sa (R.N.); 202007772@stu.jazanu.edu.sa (G.A.A.)

**Keywords:** electronic cigarettes, sleep quality, PSQI, young adults, Saudi Arabia, Jazan

## Abstract

**Purpose**: This study aimed to assess the association between electronic cigarette use and sleep quality among young adults aged 18 to 25 years. **Methods**: This cross-sectional study was conducted in the Jazan region using a structured, self-administered questionnaire. Sleep quality was assessed using the Pittsburgh Sleep Quality Index (PSQI), with global scores >5 indicating poor sleep quality. Smoking behaviors, including electronic and combustible cigarette use, along with demographic, socioeconomic, and clinical variables, were collected. Multivariable generalized linear modeling was used to identify factors independently associated with PSQI global scores. **Results**: Among 423 participants (median [IQR] age, 21.0 [20.0–24.0] years), 51.3% were classified as having poor sleep quality (PSQI > 5). In univariate analysis, male, electronic cigarette use, and presence of comorbidity were significantly associated with poor sleep quality. Electronic cigarette users had a higher prevalence of poor sleep compared with nonusers (64.0% vs. 46.6%; *p* = 0.01). In multivariable logistic regression, electronic cigarette use remained independently associated with increased odds of poor sleep (odds ratio [OR], 2.86; 95% CI, 1.31–6.24; *p* = 0.008), whereas combustible cigarette use was not significant. **Conclusions**: In this study of young adults in Jazan, poor sleep quality was common and was associated with electronic cigarette use and comorbidity burden, whereas combustible cigarette use was not associated after adjustment. These findings suggest that vaping may be an important correlate of sleep health in this population. Further longitudinal studies are needed to clarify the temporal relationship between electronic cigarette use and sleep outcomes.

## 1. Introduction

Electronic cigarette use has emerged as a significant public health concern among adolescents and young adults, driven by rapid uptake and widespread perceptions of reduced harm despite limited evidence on long-term health effects [1,2]. In Saudi Arabia, this concern is particularly pronounced, as recent national and regional studies indicate that vaping is highly prevalent among younger populations and may rival or exceed experimentation with combustible cigarettes in some student groups [3]. Evidence from Riyadh and Jazan further demonstrates substantial use, favorable attitudes toward vaping, and notable levels of dependence, underscoring the need for context-specific investigations beyond prevalence estimates [3,4,5].

Sleep health represents a critical outcome in this population. Young adulthood is characterized by academic and occupational transitions, irregular routines, high digital engagement, and increasing nicotine exposure, all of which may adversely affect sleep timing, duration, and quality. Poor sleep has been consistently associated with impaired cognitive performance, mood disturbances, reduced academic productivity, and adverse cardiometabolic outcomes [6,7]. Nicotine exposure is biologically plausible as a contributor to sleep disturbance, given its stimulant properties, potential to delay sleep onset, increase nocturnal arousal, and disrupt sleep architecture [8]. Sleep quality is influenced by multiple behavioral, psychological, and lifestyle factors, including mental health status and physical activity, which may interact with tobacco-related behaviors and contribute to sleep disturbances [6,7,8].

Accumulating international evidence suggests an association between electronic cigarette use and impaired sleep outcomes. Studies among young adults have demonstrated that vaping is associated with poorer sleep health, including shorter sleep duration and increased sleep complaints, even among nondaily users [9,10,11]. Analyses of large population-based datasets have further shown that electronic cigarette users, combustible cigarette smokers, and dual users exhibit higher odds of inadequate sleep, with the greatest burden observed among dual users [9]. While previous studies have reported associations between electronic cigarette use and sleep outcomes, evidence from Saudi Arabia remains limited, and important regional differences in demographic, behavioral, and sociocultural factors warrant further investigation [9,10,11,12].

In Saudi Arabia, the evolving epidemiology of tobacco and vaping behaviors strengthens the need for further investigation. Recent studies highlight increasing experimentation, misperceptions of harm, and substantial dependence among young users [3,4,5]. Local evidence also demonstrates that combustible cigarette use is associated with poorer sleep quality, as measured by the Pittsburgh Sleep Quality Index (PSQI), supporting the biological and behavioral plausibility of a similar relationship for electronic cigarettes [3,13]. Despite this emerging evidence, important gaps remain. Existing Saudi studies are often limited to university-based samples, heterogeneous exposure definitions, and narrow outcome measures, restricting comparability and generalizability [2,3,4,5]. Moreover, community-based evidence examining electronic cigarette use and sleep quality remains scarce, particularly in the Jazan region, where a previous study has documented notable tobacco-use behaviors among young adults university students [5], highlighting the importance of examining vaping-related health outcomes within this local context. Generating region-specific evidence is essential to inform targeted prevention strategies, educational interventions, and tobacco control policies.

Against this background, this study aimed to evaluate the association between electronic cigarette use and sleep quality among young adults aged 18 to 25 years in Jazan, Saudi Arabia, using the Pittsburgh Sleep Quality Index. We further compared sleep outcomes across electronic cigarette and combustible cigarette use. We hypothesized that electronic cigarette use would be associated with poorer sleep quality, reflected by higher global PSQI scores, and that this association would persist after adjustment for sociodemographic factors and comorbidity burden.

## 2. Methods

### 2.1. Study Design and Setting

This analytical cross-sectional study examined the association between electronic cigarette use and sleep quality among young adults aged 18 to 25 years in the Jazan region of Saudi Arabia. The study was conducted and reported in accordance with established guidelines for observational epidemiological studies [14]. Jazan is located in southwestern Saudi Arabia and has an estimated population of approximately 1.4 million across 13 administrative subregions.

### 2.2. Study Population

The target population included young adults aged 18 to 25 years residing in Jazan. Eligible participants were required to be able to read and understand Arabic and to provide informed consent. Individuals younger than 18 years or older than 25 years, non-Arabic speakers, illiterate individuals, and those unwilling to participate were excluded. Participants with incomplete data on key variables, including smoking status or sleep outcomes, were excluded from the final analysis. Participants reporting concurrent use of electronic cigarettes and combustible cigarettes were excluded because of the limited number of dual users and the difficulty in attributing observed associations to a specific tobacco product exposure.

### 2.3. Sampling and Sample Size

A nonprobability convenience sampling approach was used. Data were collected through a web-based questionnaire developed using Google Forms and distributed via social media platforms and community and student networks to maximize reach within the target population. The minimum required sample size was calculated using a single population proportion formula with a 95% confidence level, 50% assumed prevalence, and a 5% margin of error, yielding a minimum sample size of 384 participants. Although the sample size calculation was based on estimating a population proportion, the final sample size and number of outcome events were also sufficient for the planned multivariable regression analyses, with the events-per-variable ratio exceeding commonly recommended thresholds for model stability and reliable parameter estimation.

### 2.4. Data Collection and Instrument

The study questionnaire consisted of three sections: (1) demographic and clinical characteristics, (2) smoking behaviors, and (3) sleep quality assessment. The study-specific questionnaire items (demographic, clinical, and smoking-related questions) were originally developed in English and underwent forward and backward translation by bilingual experts to ensure linguistic and conceptual equivalence. A pilot study was conducted to assess clarity, comprehension, and cultural appropriateness, and minor revisions were made before full-scale data collection. Sleep quality was assessed using the previously validated Arabic version of the Pittsburgh Sleep Quality Index (PSQI).

### 2.5. Measures and Variable Definitions

The primary outcome was sleep quality, assessed using the previously validated Arabic version of the Pittsburgh Sleep Quality Index (PSQI). The questionnaire consisted of three sections: sociodemographic characteristics, tobacco-use behaviors, and sleep quality assessment using the previously validated Arabic version of the Pittsburgh Sleep Quality Index (PSQI). The tobacco-use questions were adapted from previously published tobacco-use surveys and modified to align with the objectives of the study [15,16,17]. Prior to implementation, the questionnaire was pilot tested among 7 participants to assess clarity, readability, and completion time, and minor modifications were made based on participant feedback. The primary outcome was sleep quality, assessed using the Pittsburgh Sleep Quality Index (PSQI), a validated instrument measuring sleep quality over the preceding month across seven domains: subjective sleep quality, sleep latency, sleep duration, habitual sleep efficiency, sleep disturbances, use of sleep medication, and daytime dysfunction. Each component is scored from 0 to 3, yielding a global score ranging from 0 to 21, with higher scores indicating poorer sleep quality. A global PSQI score greater than 5 was used to define poor sleep quality, consistent with established thresholds [15,16,17].

Smoking exposure was assessed using self-reported use of electronic cigarettes and combustible cigarettes. Current use was defined as any use of the product within the past 30 days, consistent with standard epidemiologic practice in tobacco research [18,19]. Participants were classified separately for electronic cigarette use and combustible cigarette use as current users or non-current users. Non-current users included individuals who reported no use within the past 30 days, regardless of prior experimentation. This definition was used to distinguish recent active exposure from past or experimental use and to improve comparability with previous population-based smoking studies. Individuals reporting dual use were excluded from subgroup comparisons to reduce misclassification and confounding [20]. Demographic variables included age, sex, educational level, marital status, monthly income, and employment status. Participants were classified as having a comorbidity if they self-reported a physician diagnosis of at least one chronic health condition. These conditions included diabetes mellitus, hypertension, cardiovascular disease, respiratory diseases (e.g., asthma or chronic respiratory conditions), and psychological disorders (e.g., depression or anxiety). The PSQI was used in accordance with the official conditions of use for non-commercial academic research, and the original instrument and scoring methodology were applied without modification.

### 2.6. Validity and Reliability

The validated Arabic version of the Pittsburgh Sleep Quality Index (PSQI) was used in accordance with the official conditions governing its use for non-commercial academic research. The original instrument and scoring methodology were applied without modification, and the original publication was appropriately cited. Content validity was supported through the use of validated instruments and established survey items. Sleep quality was assessed using the previously validated Arabic version of the Pittsburgh Sleep Quality Index (PSQI), which has demonstrated acceptable psychometric properties in Arabic-speaking populations. The PSQI was used in accordance with its official conditions of use for non-commercial academic research, and the original instrument and scoring methodology were applied without modification. Internal consistency in the present study was good (Cronbach’s α = 0.80) [21].

### 2.7. Bias and Ethics

Potential sources of bias include selection bias due to nonprobability sampling and information bias resulting from self-reported measures. Recall and social desirability bias may have influenced reporting of smoking behaviors and sleep patterns. These were mitigated through the use of standardized and validated instruments.

Ethical approval was obtained from the Jazan Health Ethics Committee. Participation was voluntary, and informed consent was obtained from all participants. Data were collected anonymously and used solely for research purposes.

### 2.8. Statistical Analysis

All analyses were conducted using SAS version 9.4. A 2-sided *p*-value < 0.05 was considered statistically significant. Categorical variables were compared using χ^2^ tests, and age was compared between sleep-quality groups using an independent samples *t*-test. The PSQI global score was analyzed as both a continuous variable and a binary outcome (≤5 vs. >5).

### 2.9. Multivariable Analysis

Multivariable logistic regression was used to identify factors independently associated with poor sleep quality (PSQI > 5). The primary exposure variables were current electronic cigarette use and current combustible cigarette use. The multivariable model adjusted for age, sex, educational level, marital status, monthly income, employment status, and comorbidity status.

Smoking exposures were modeled as separate binary variables to allow independent estimation of their associations with sleep outcomes. Adjusted odds ratios (ORs) and 95% confidence intervals (CIs) were reported.

### 2.10. Model Diagnostics

Model assumptions were assessed prior to interpretation. Linearity in the logit for continuous variables was evaluated using fractional polynomials and graphical assessment. Multicollinearity was assessed using variance inflation factors, with no evidence of significant collinearity [22]. Model calibration was evaluated using the Hosmer–Lemeshow test, and discrimination was assessed using the area under the receiver operating characteristic curve. Influential observations were assessed using leverage statistics and Cook’s distance, with no evidence of undue influence [23]. Model diagnostics showed acceptable discrimination (AUC = 0.708) and adequate calibration using the Hosmer–Lemeshow test (χ^2^ = 12.81, df = 8, *p* = 0.118). Linearity in the logit for age was supported, and no severe multicollinearity or influential observations were identified. see Tables S1–S4, Figures S1–S5 in the Supplementary Materials.

## 3. Results

### 3.1. Participant Characteristics

A total of 564 responses were received through the web-based questionnaire. After exclusion of incomplete questionnaires and responses with missing key analytical variables, 423 participants were included in the final analysis, corresponding to an overall completion rate of approximately 75%. Missingness in the key analytical variables did not demonstrate an apparent systematic pattern. The median (IQR) age was 21.0 (20.0–24.0) years. Most participants were Saudi (386 [91.3%]) and male (236 [55.8%]). Regarding educational attainment, over half of participants were undergraduate students (223 [52.7%]), while 196 (46.3%) had a high school education or less and 4 (0.9%) reported no formal college education. The majority of participants were single (345 [81.6%]), with smaller proportions married (72 [17.0%]) or divorced (6 [1.4%]). Monthly income was predominantly less than 3000 SAR (274 [64.8%]), with the remaining participants distributed across higher income categories, including 41 (9.7%) earning 3000–4999 SAR, 33 (7.8%) earning 5000–8999 SAR, 37 (8.7%) earning 9000–15,000 SAR, and 38 (9.0%) earning more than 15,000 SAR. Most participants were students (268 [63.4%]), while 95 (22.5%) were unemployed and 60 (14.2%) were employed. Regarding smoking behaviors, 114 participants (27.0%) reported being current electronic cigarette users, whereas 96 (22.7%) reported being current combustible cigarette users.

**Prevalence of self-reported clinical comorbidities (Table 1).**

**Figure 1:**
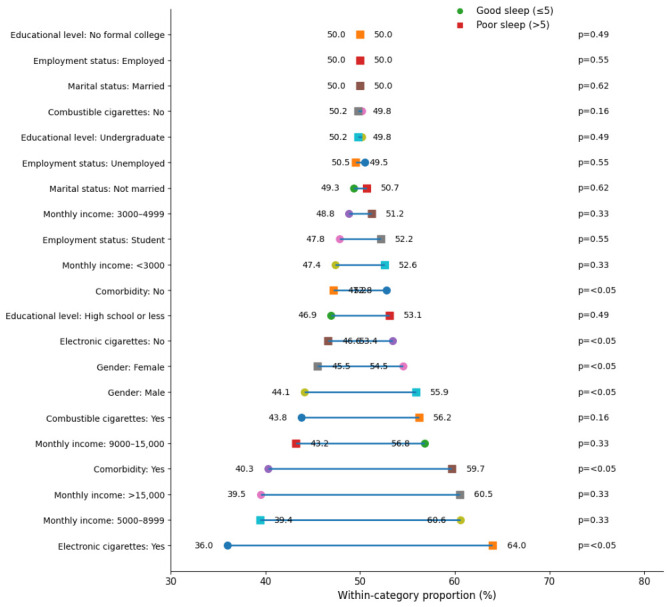
Univariate distribution of sleep quality (PSQI global score) across demographic and behavioral characteristics. **Footnote**: Values represent within-category proportions (%) of participants classified as good sleep (PSQI ≤ 5) and poor sleep (PSQI > 5). Lines connect proportions within each category to illustrate differences in distribution. *p*-values were derived using Pearson χ^2^ tests comparing sleep quality categories across each variable. PSQI indicates Pittsburgh Sleep Quality Index.

In univariate analysis, **female sex, electronic cigarette use, and presence of comorbidity** were significantly associated with poorer sleep quality. Male participants had a higher proportion of poor sleep compared with females (55.9% vs. 45.5%; *p* = 0.02). Electronic cigarette users had a substantially higher proportion of poor sleep (64.0%) compared with non-users (46.6%) (*p* = 0.01). Similarly, participants with at least one comorbid condition showed worse sleep quality (59.7% poor sleep) compared with those without comorbidities (47.2%) (*p* = 0.03). No other variables demonstrated statistically significant associations (Table 1, Figure 1).

### 3.2. Sleep Quality and PSQI Components

**Figure 2:**
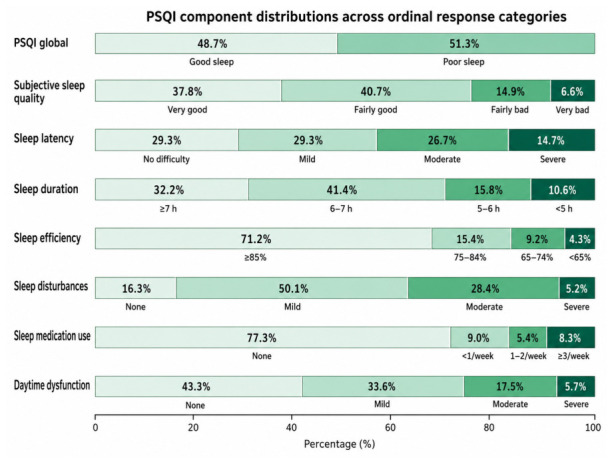
Distribution of Pittsburgh Sleep Quality Index (PSQI) Components Across Ordinal Response Categories.

Overall, 217 participants (51.3%) were classified as having poor sleep quality (PSQI > 5), while 206 (48.7%) had good sleep quality. For subjective sleep quality, most participants reported fairly good (172 [40.7%]) or very good sleep (160 [37.8%]), whereas 91 (21.5%) reported fairly bad or very bad sleep. Sleep latency was commonly impaired, with 175 participants (41.4%) reporting moderate-to-severe difficulty initiating sleep. Regarding sleep duration, the majority reported sleeping 6–7 h (175 [41.4%]), while 112 (26.4%) reported less than 6 h per night. Sleep efficiency was generally preserved, with 301 participants (71.2%) achieving ≥85% efficiency. Sleep disturbances were highly prevalent, with 354 participants (83.7%) reporting at least mild disturbances. In contrast, most participants reported no use of sleep medications (327 [77.3%]). Daytime dysfunction was also common, with 240 participants (56.7%) reporting at least mild impairment (Table 2, Figure 2).

### 3.3. Partner-Reported Sleep Issues

**Figure 3:**
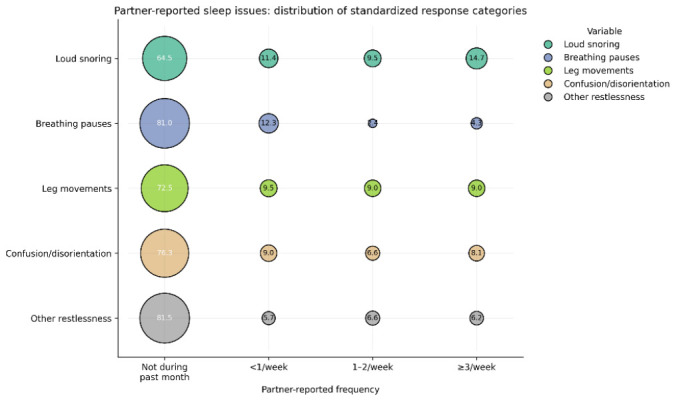
Partner-reported sleep issues: distribution across standardized frequency categories. **Footnote:** Bubble size represents the proportion of participants reporting each symptom within standardized frequency categories over the past month. Larger bubbles indicate higher prevalence. Frequencies were categorized as not during the past month, <1/week, 1–2/week, and ≥3/week. Colors correspond to specific symptoms, including loud snoring, breathing pauses, leg movements, confusion/disorientation, and other restlessness. Percentages displayed within bubbles reflect the proportion of responses within each category. In addition to the standard PSQI components, participants were asked to report whether a bed partner or roommate had observed selected sleep-related behaviors, including loud snoring, breathing pauses during sleep, leg twitching, or episodes of confusion. These responses were self-reported by participants and were not obtained directly from bed partners or roommates. In the Saudi context, where many young adults live within family households or shared living environments, these descriptive items were retained to provide additional context on observable sleep-related disturbances.

Partner-reported sleep disturbances were reported infrequently, with most participants reporting no symptoms during the past month across all domains. Absence of symptoms was most commonly reported for breathing pauses (81.0%), other restlessness (81.5%), confusion or disorientation (76.3%), and leg movements (72.5%), while loud snoring was absent in 64.5% of participants. Loud snoring was the most frequently reported symptom, with 35.5% of participants experiencing it at least once during the past month, including 14.7% occurring three or more times per week. Other sleep-related disturbances were less frequent, with moderate-to-high frequency (≥1–2 times per week) ranging from approximately 8% to 15% across symptom domains. Overall, these findings indicate that partner-observed sleep disturbances were present in a subset of participants but were generally reported at low frequencies, with frequent symptoms being uncommon (Figure 3).

### 3.4. Multivariable Logistic Regression Analysis

In multivariable logistic regression analysis, electronic cigarette use was associated with higher odds of poor sleep quality (odds ratio [OR], 2.86; 95% CI, 1.31–6.24; *p* = 0.008). Comorbidity was also associated with increased odds of poor sleep (OR, 1.72; 95% CI, 1.01–2.93; *p* = 0.045). In contrast, combustible cigarette use was not associated with sleep quality (OR, 1.86; 95% CI, 0.79–4.39; *p* = 0.16). Male sex was associated with lower odds of poor sleep compared with female sex (OR, 0.41; 95% CI, 0.20–0.85; *p* = 0.02). Age was not associated with sleep quality (OR per 1-year increase, 1.04; 95% CI, 0.96–1.13; *p* = 0.29). Among socioeconomic factors, participants with a monthly income of 5000–8999 SAR had lower odds of poor sleep compared with those earning less than 3000 SAR (OR, 0.16; 95% CI, 0.03–0.79; *p* = 0.03), whereas other income categories were not associated with the outcome. Educational level, marital status, and employment status were not associated with sleep quality (Table 3).

## 4. Discussion

In this cross-sectional study of young adults in Jazan, Saudi Arabia, poor sleep quality was highly prevalent, affecting more than half of participants, indicating a substantial burden of sleep disturbance in early adulthood. Sleep impairment was not limited to the global PSQI score but was consistently observed across multiple domains, particularly sleep latency, sleep disturbances, and daytime dysfunction, reflecting a multidimensional pattern of sleep disruption in this population. Electronic cigarette use emerged as the most consistent and clinically relevant factor associated with poor sleep quality. This association remained robust after adjustment, with users demonstrating nearly threefold higher odds of poor sleep. These findings align with international evidence showing that e-cigarette use is associated with shorter sleep duration, increased sleep disturbances, and poorer overall sleep health among young adults [11,24]. Population-based analyses from the United States have similarly reported higher odds of inadequate sleep among e-cigarette users compared with nonusers, independent of traditional risk factors [25]. The biological plausibility of this relationship is well established, as nicotine exerts stimulant effects on the central nervous system, delays sleep onset, and disrupts normal sleep architecture [24]. The observed association is consistent with findings from a recent systematic review, which reported that electronic cigarette use was associated with poorer sleep health, including shorter sleep duration, increased insomnia symptoms, and reduced sleep quality across multiple study populations [10].

In contrast, combustible cigarette use was not independently associated with sleep quality after adjustment. This finding differs from some previous studies, including those conducted in Saudi Arabia, where conventional smoking has been linked to poorer PSQI scores and increased sleep disturbances [26]. However, attenuation of this association after adjustment has also been reported in international studies, suggesting that the relationship between combustible smoking and sleep may be partially mediated by comorbidities or correlated behavioral factors [27]. Unlike combustible cigarettes, electronic cigarettes may permit more frequent and intermittent nicotine use throughout the day because they are easier to access and can be used in a wider range of settings. This pattern of nicotine exposure may contribute to sustained physiological stimulation and has been associated with adverse sleep outcomes through nicotine’s effects on arousal pathways, sleep architecture, and circadian regulation [9,10].

The role of comorbidity further underscores the multifactorial nature of sleep impairment. Participants with at least one chronic condition demonstrated poorer sleep in univariate analysis, consistent with extensive literature linking chronic diseases, particularly respiratory and psychological conditions, to sleep disturbances [27,28]. Although this association was not retained in multivariable models, it likely reflects shared pathways between underlying health status and behavioral exposures, including nicotine use. The overall high prevalence of sleep disturbances observed in this cohort is consistent with both regional and international findings demonstrating substantial sleep impairment among young adults, even in the absence of severe clinical disease [19,25].

Although crude sleep-quality distributions differed between males and females, the multivariable analysis indicated that female sex was independently associated with poorer sleep quality after adjustment for potential confounding factors. This finding should therefore be interpreted in the context of the adjusted model rather than the unadjusted descriptive comparisons. Female sex was independently associated with poorer sleep quality in the adjusted model. However, this finding should be interpreted as an observational association rather than evidence of sex-specific differences in the relationship between tobacco use and sleep quality. This pattern has been consistently documented across diverse populations and is often attributed to a combination of hormonal, psychological, and social determinants influencing sleep quality [29]. Similarly, the observed association between moderate income and better sleep outcomes is consistent with evidence suggesting that socioeconomic status influences sleep through pathways related to stress, living conditions, and access to health-promoting resources, although such relationships are often nonlinear and context-specific [29].

From a public health perspective, these findings are particularly relevant in Saudi Arabia, where electronic cigarette use has increased rapidly among adolescents and young adults. Recent studies from Jazan and other regions have documented high prevalence and increasing dependence on vaping products in this population [2,3,5]. The present study extends this evidence by demonstrating a clear association between electronic cigarette use and poor sleep quality, an outcome with important implications for mental health, academic performance, and long-term cardiometabolic risk. These findings underscore the need for integrated public health interventions targeting both nicotine use, particularly vaping and sleep health as interrelated determinants of well-being in young populations. Overall, this study demonstrates that sleep impairment among young adults is common and appears to be strongly associated with modifiable behavioral factors, particularly electronic cigarette use, in addition to underlying health status. These findings are consistent with and extend the growing global literature emphasizing the importance of addressing emerging nicotine behaviors and sleep health concurrently within public health strategies.

### Limitations

Several limitations should be considered when interpreting these findings. First, the cross-sectional design precludes causal inference, and the directionality of the association between electronic cigarette use and poor sleep cannot be established. Reverse causation is possible, whereby individuals with poor sleep may be more likely to use nicotine products. Second, the use of self-reported data introduces the potential for recall and social desirability bias, particularly in reporting smoking behaviors and sleep patterns. Third, the use of convenience sampling and online recruitment may limit generalizability, as the study sample may not fully represent the broader young adult population in Jazan. Fourth, residual confounding cannot be excluded, particularly for unmeasured factors such as caffeine intake, screen exposure, mental health status, and physical activity. Finally, the use of a composite comorbidity variable, while capturing overall disease burden, may obscure the differential effects of specific conditions on sleep outcomes.

## 5. Conclusions

In this cross-sectional study of young adults in Jazan, Saudi Arabia, poor sleep quality was common and was associated with electronic cigarette use and comorbidity burden, whereas combustible cigarette use was not associated after adjustment. These findings suggest that vaping may be an important correlate of sleep health in this population. Further longitudinal studies are needed to clarify the temporal relationship between electronic cigarette use and sleep outcomes.

## Figures and Tables

**Table 1:** Participant Characteristics and Their Association with Sleep Quality (PSQI > 5).

Variable	Category	Overall N (%)	Good Sleep (≤5), n (%)	Poor Sleep (>5), n (%)	*p* Value
**Age, years**	Median (IQR)	—	21.0 (20.0–24.0)	21.0 (20.0–24.0)	0.29 †
**Gender**	Female	187 (44.2)	102 (54.5)	85 (45.5)	0.02
	Male	236 (55.8)	104 (44.1)	132 (55.9)	
**Educational level**	Undergraduate	223 (52.7)	112 (50.2)	111 (49.8)	0.49
	High school or less	196 (46.3)	92 (46.9)	104 (53.1)	
	No formal college	4 (0.9)	2 (50.0)	2 (50.0)	
**Marital status**	Not married *	345 (81.6)	170 (49.3)	175 (50.7)	0.62
	Married	72 (17.0)	36 (50.0)	36 (50.0)	
**Monthly income (SAR)**	<3000	274 (64.8)	130 (47.4)	144 (52.6)	0.33
	3000–4999	41 (9.7)	20 (48.8)	21 (51.2)	
	5000–8999	33 (7.8)	20 (60.6)	13 (39.4)	
	9000–15,000	37 (8.7)	21 (56.8)	16 (43.2)	
	>15,000	38 (9.0)	15 (39.5)	23 (60.5)	
**Employment status**	Student	268 (63.4)	128 (47.8)	140 (52.2)	0.55
	Unemployed	95 (22.5)	48 (50.5)	47 (49.5)	
	Employed	60 (14.2)	30 (50.0)	30 (50.0)	
**Comorbidity**	No	284 (67.1%)	150 (52.8)	134 (47.2)	0.03
	Yes	139(32.9%)	56 (40.3)	83 (59.7)	
**Electronic cigarettes (current user)**	No	309 (73.0)	165 (53.4)	144 (46.6)	0.01
	Yes	114 (27.0)	41 (36.0)	73 (64.0)	
**Combustible cigarettes (current user)**	No	327 (77.3)	164 (50.2)	163 (49.8)	0.16
	Yes	96 (22.7)	42 (43.8)	54 (56.2)	

Data are presented as n (%) unless otherwise indicated. Percentages in the Good Sleep and Poor Sleep columns are calculated within each row/category to show the distribution of sleep quality within each demographic, clinical, or behavioral characteristic. *p* values were derived using χ^2^ tests for categorical variables. † Age was compared using an independent samples *t*-test. * Not married includes single, widowed, divorced, or separated.

**Table 2:** Distribution of PSQI Component Scores by Ordinal Categories (N = 423).

Variable	Category	N (%)
**PSQI global**	≤5 (Good sleep)	206 (48.7)
	>5 (Poor sleep)	217 (51.3)
**Subjective sleep quality**	Very good (0)	160 (37.8)
	Fairly good (1)	172 (40.7)
	Fairly bad (2)	63 (14.9)
	Very bad (3)	28 (6.6)
**Sleep latency**	No difficulty (0)	124 (29.3)
	Mild (1)	124 (29.3)
	Moderate (2)	113 (26.7)
	Severe (3)	62 (14.7)
**Sleep duration**	≥7 h (0)	136 (32.2)
	6–7 h (1)	175 (41.4)
	5–6 h (2)	67 (15.8)
	<5 h (3)	45 (10.6)
**Sleep efficiency**	≥85% (0)	301 (71.2)
	75–84% (1)	65 (15.4)
	65–74% (2)	39 (9.2)
	<65% (3)	18 (4.3)
**Sleep disturbances**	None (0)	69 (16.3)
	Mild (1)	212 (50.1)
	Moderate (2)	120 (28.4)
	Severe (3)	22 (5.2)
**Sleep medication use**	None (0)	327 (77.3)
	<1/week (1)	38 (9.0)
	1–2/week (2)	23 (5.4)
	≥3/week (3)	35 (8.3)
**Daytime dysfunction**	None (0)	183 (43.3)
	Mild (1)	142 (33.6)
	Moderate (2)	74 (17.5)
	Severe (3)	24 (5.7)

**Table 3:** Multivariable Logistic Regression of Factors Associated with Poor Sleep Quality (PSQI > 5) (N = 423).

Variable	Category	Odds Ratio (OR)	95% CI	*p* Value
**Age, years**	Per 1-year increase	1.04	0.96–1.13	0.29
**Gender**	Female	Reference	—	—
	Male	**0.41**	**0.20–0.85**	**0.018**
**Educational level**	Undergraduate	Reference	—	—
	High school or less	1.27	0.64–2.50	0.49
	No formal college	1.51	0.04–56.9	0.83
**Marital status**	Not married	Reference	—	—
	Married	1.19	0.59–2.41	0.62
**Monthly income (SAR)**	<3000	Reference	—	—
	3000–4999	0.56	0.17–1.82	0.33
	5000–8999	**0.16**	**0.03–0.79**	**0.025**
	9000–15,000	0.26	0.06–1.24	0.091
	>15,000	0.59	0.13–2.80	0.51
**Employment status**	Student	Reference	—	—
	Unemployed	1.35	0.50–3.67	0.55
	Employed	0.85	0.29–2.51	0.77
**Comorbidity (≥1 condition)**	No	Reference	—	—
	Yes	**1.72**	**1.01–2.93**	**0.045**
**Current electronic cigarettes**	No	Reference	—	—
	Yes	**2.86**	**1.31–6.24**	**0.008**
**Current combustible cigarettes**	No	Reference	—	—
	Yes	1.86	0.79–4.39	0.16

Data are presented as odds ratios (ORs) and 95% confidence intervals (CIs) from multivariable logistic regression. The outcome was poor sleep quality (PSQI global score > 5). All variables were entered simultaneously. Reference categories are shown for each categorical variable.

## Data Availability

The datasets generated and analyzed during the current study are not publicly available due to institutional data-sharing restrictions but are available from the corresponding author on reasonable request.

## Supplementary Materials

The following supporting information can be downloaded at: https://www.mdpi.com/article/10.3390/healthcare14142068/s1, Table S1. Summary of Model Diagnostics; Table S2. Adjusted Logistic Regression Estimates; Table S3. Hosmer-Lemeshow Calibration Deciles; Table S4. Variance Inflation Factors; Figure S1. Receiver Operating Characteristic Curve; Figure S2. Calibration Plot; Figure S3. Multicollinearity Assessment; Figure S4. Influence and Residual Diagnostics; Figure S5. Predictor Correlation Matrix.

## References

[1] Rodrigo C.S., Escolar-Llamazares M.C., Val E.I.M. (2026). Adolescents’ perceptions of tobacco and electronic cigarette prevention: Six school-based cohorts (2010/11–2023/24). Addict. Behav. Rep..

[2] Aqeeli A., Alsabaani A.A., Alshaiban H., Alqassim A.Y., Alahmar A.S., Sabai A., Alwadani S. (2024). Assessing dependence and perceptions of harm of electronic cigarettes among Saudi university students: A cross-sectional study. Healthcare.

[3] AlHumaidan N.I., AlZelfawi L.A., AlHindawi Z.A., AlDosari L.M., AlTowaijri A.M., AlFaisal N.F. (2024). Prevalence, perception, and attitude regarding electronic cigarettes usage among young adults in Riyadh, Saudi Arabia: A cross-sectional study. Saudi Med. J..

[4] AlDukhail S.K., El Desouky E.D., Monshi S.S., Al-Zalabani A.H., Alanazi A.M., El Dalatony M.M. (2025). Electronic cigarette use among adolescents in Saudi Arabia: A national study. Tob. Induc. Dis..

[5] Somaili M., Mahfouz M.S., Elmakk E.E.H., Mohamed A.H., Mosleh A.A., Alshekh F.O., Alharbi A.I. (2025). Prevalence of gastroesophageal reflux disease among university students who smoke electronic cigarettes in Jazan, Saudi Arabia. Saudi J. Health Sci..

[6] Dugas E.N., Sylvestre M.P., O’Loughlin E.K., Brunet J., Kakinami L., Constantin E., O’Loughlin J. (2017). Nicotine dependence and sleep quality in young adults. Addict. Behav..

[7] Brett E.I., Miller M.B., Leavens E.L., Lopez S.V., Wagener T.L., Leffingwell T.R. (2020). Electronic cigarette use and sleep health in young adults. J. Sleep Res..

[8] So C.J., Meers J.M., Alfano C.A., Garey L., Zvolensky M.J. (2021). Main and interactive effects of nicotine product type on sleep health among dual combustible and e-cigarette users. Am. J. Addict..

[9] Wang S., Nandy R.R., Rossheim M.E. (2024). Associations between e-cigarette use and sleep health among adults in the United States, NHANES 2015–2018. Sleep Med..

[10] Sulthana H., Jan A., Verma A., Sah R., Mehta R., Ullah A., Ullah A. (2025). Impact of electronic cigarette use and sleep duration, sleep issues and insomnia: A systematic review and meta-analysis. Front. Public Health.

[11] Riehm K.E., Rojo-Wissar D.M., Feder K.A., Mojtabai R., Spira A.P., Thrul J., Crum R.M. (2019). E-cigarette use and sleep-related complaints among youth. J. Adolesc..

[12] Merianos A.L., Mahabee-Gittens E.M., Hill M.J., Olaniyan A.C., Smith M.L., Choi K. (2023). Electronic cigarette use and cigarette smoking associated with inadequate sleep duration among US young adults. Prev. Med..

[13] Al-Mshari A., AlSheikh M.H., Latif R., Mumtaz S., Albaker W., Al-Hariri M. (2022). Impact of smoking intensities on sleep quality in young Saudi males: A comparative study. J. Med. Life.

[14] Von Elm E., Altman D.G., Egger M., Pocock S.J., Gøtzsche P.C., Vandenbroucke J.P. (2007). The Strengthening the Reporting of Observational Studies in Epidemiology (STROBE) statement: Guidelines for reporting observational studies. Lancet.

[15] Al Maqbali M., Hughes C., Gracey J., Rankin J., Dunwoody L., Hacker E. (2020). Validation of the Pittsburgh Sleep Quality Index (PSQI) with Arabic cancer patients. Sleep Biol. Rhythms.

[16] Buysse D.J., Reynolds C.F., Monk T.H., Berman S.R., Kupfer D.J. (1989). The Pittsburgh Sleep Quality Index: A new instrument for psychiatric practice and research. Psychiatry Res..

[17] Huang Y., Zhu M. (2020). Increased global PSQI score is associated with depressive symptoms in an adult population from the United States. Nat. Sci. Sleep.

[18] Jia Y., Chen S., Deutz N.E., Bukkapatnam S.T., Woltering S. (2019). Examining the structural validity of the Pittsburgh Sleep Quality Index. Sleep Biol. Rhythms.

[19] Birdsey J. (2023). Tobacco product use among US middle and high school students—National Youth Tobacco Survey, 2023. MMWR Morb. Mortal. Wkly. Rep..

[20] Gustafson P., Greenland S. (2025). Misclassification. Handbook of Epidemiology.

[21] Adamson K.A., Prion S. (2013). Reliability: Measuring internal consistency using Cronbach’s α. Clin. Simul. Nurs..

[22] Tukur K., Usman A.U. (2016). Binary logistic regression analysis. J. Curr. Res..

[23] Senaviratna N.A.M.R., Cooray T.M.J.A. (2019). Diagnosing multicollinearity of logistic regression model. Asian J. Probab. Stat..

[24] Dani J.A., Bertrand D. (2007). Nicotinic acetylcholine receptors and nicotinic cholinergic mechanisms of the central nervous system. Annu. Rev. Pharmacol. Toxicol..

[25] Poudel L., Baral A., Abdshah A., Grealis K., Aka A., Paudel S., Vidot D.C. (2025). Sleep disturbances among young adult dual users of cigarettes and e-cigarettes: Analysis of the 2020 National Health Interview Survey. PLoS ONE.

[26] AlRyalat S.A., Kussad S., El Khatib O., Hamad I., Al-Tanjy A., Alshnneikat M., AbuMahfouz B. (2021). Assessing the effect of nicotine dose in cigarette smoking on sleep quality. Sleep Breath.

[27] Lee S.H., Kim K.U., Lee H., Park H.K., Kim Y.S., Lee M.K. (2019). Sleep disturbance in patients with mild-to-moderate chronic obstructive pulmonary disease. Clin. Respir. J..

[28] Aldabayan Y.S., Alqahtani J.S., Al Rajeh A.M., Abdelhafez A.I., Siraj R.A., Thirunavukkarasu V., Aldhahir A.M. (2022). Prevalence and predictors of sleep disturbance, anxiety and depression among patients with chronic respiratory diseases. Int. J. Environ. Res. Public Health.

[29] Lee H.W. (2024). Sex/gender differences in sleep physiology and sleep disorders. Sex/Gender-Specific Medicine in Clinical Areas.

