# Supplementary material for: Electronic Cigarette Use and Sleep Quality Among Young Adults in Jazan, Saudi Arabia: A Community-Based Cross-Sectional Study
Source: Healthcare (Basel). 2026 Jul 10;14(14):2068. doi: 10.3390/healthcare14142068 (PMC13411711; doi:10.3390/healthcare14142068)
Supplement: Supplementary file 1 [file healthcare-14-02068-s001.zip › healthcare-4299205-supplementary.pdf]

Supplementary File: Adjusted Logistic Regression Model Diagnostics

Study: Electronic Cigarette Use and Sleep Quality Among Young Adults in Jazan, Saudi Arabia

This supplementary file reports diagnostic assessments for the adjusted logistic regression model evaluating factors associated with poor sleep quality, defined as Pittsburgh Sleep Quality Index (PSQI) global score >5. The model included age, sex, educational level, marital status, monthly income, employment status, comorbidity, current electronic cigarette use, and current combustible cigarette use.

Supplementary Table S1. Summary of Model Diagnostics

Domain	Assessment	Result	Interpretation
Analytical sample	N	423	423 participants included in analysis
Outcome events	Poor sleep quality events	217	Events per parameter = 15.5
Outcome nonevents	Good sleep quality events	206	Nonevents per parameter = 14.7
Model discrimination	ROC AUC	0.708	Acceptable discrimination
Optimal threshold	Youden index threshold	0.504	Sensitivity 0.636; specificity 0.689
Calibration	Hosmer-Lemeshow test	chi-square = 12.81; df = 8; p = 0.118	No evidence of poor fit
Linearity in logit	Box-Tidwell term for age	p = 0.215	No evidence against linearity for age
Multicollinearity	Maximum VIF	1.86	No severe multicollinearity
Influence	Maximum Cook's distance	0.064	Below common threshold 4/n = 0.009
Leverage	Maximum leverage	0.303	Threshold 2p/n = 0.071
Residuals	Maximum absolute studentized residual	3.13	No systematic residual pattern observed visually

Supplementary Table S2. Adjusted Logistic Regression Estimates

Predictor	OR	Lower 95% CI	Upper 95% CI	P value
Age	1.041	0.924	1.171	0.509
Male sex	0.410	0.254	0.661	0.000
Married	0.896	0.459	1.747	0.746
Comorbidity	1.629	1.037	2.557	0.034
Current electronic cigarette use	4.038	2.252	7.242	0.000
Current combustible cigarette use	1.150	0.628	2.104	0.651
Education_High school or less	0.969	0.614	1.531	0.893
Education_No formal college	0.972	0.111	8.497	0.980
Income_3000 - 4999	1.963	0.944	4.079	0.071
Income_5000 - 8999	0.689	0.294	1.612	0.390
Income_9,000 - 15,000	0.948	0.421	2.132	0.897
Income_More than 15,000	1.601	0.755	3.393	0.220
Employment_Unemployed	0.415	0.210	0.817	0.011
Employment_Employed	0.876	0.403	1.902	0.738

Supplementary Figure S1. Receiver Operating Characteristic Curve

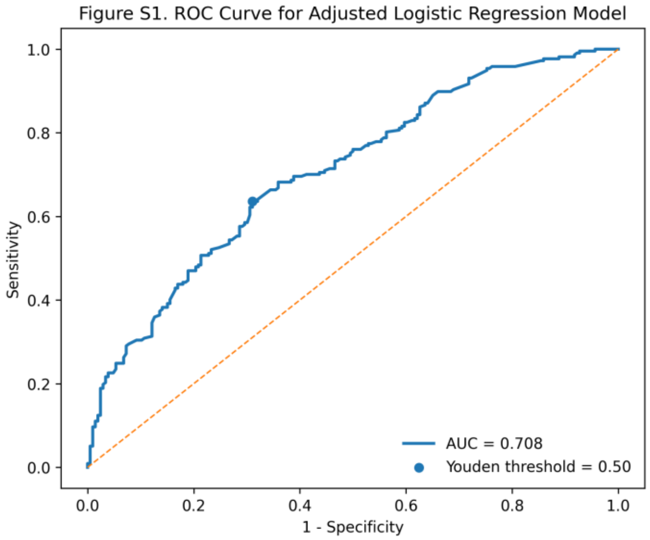

The adjusted model demonstrated acceptable discrimination (AUC = 0.708). The optimal threshold according to the Youden index was 0.504, with sensitivity 0.636 and specificity 0.689.

Supplementary Figure S2. Calibration Plot

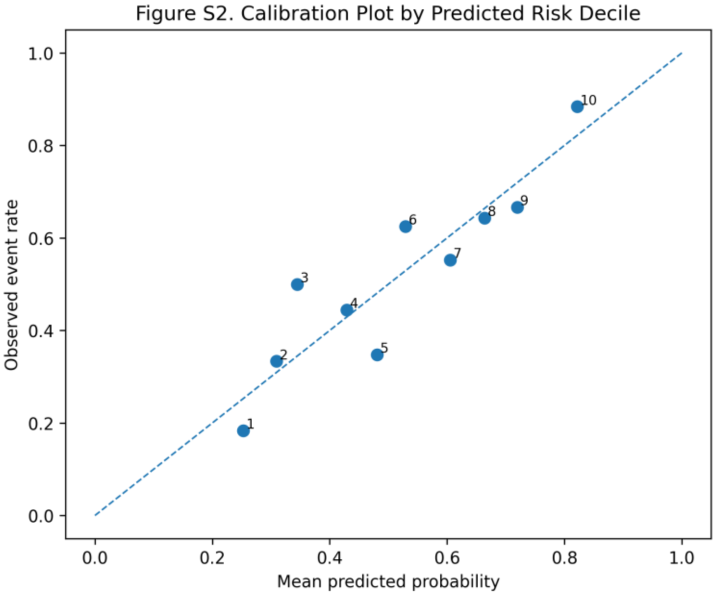

Calibration was assessed across deciles of predicted risk. The Hosmer-Lemeshow test did not indicate poor model fit (chi-square = 12.81, df = 8, p = 0.118).

Supplementary Table S3. Hosmer-Lemeshow Calibration Deciles

Risk decile	N	Observed events	Expected events	Observed event rate	Expected event rate
1.000	49.00	9.000	12.37	0.184	0.253
2.000	36.00	12.00	11.13	0.333	0.309
3.000	42.00	21.00	14.47	0.500	0.344
4.000	45.00	20.00	19.32	0.444	0.429
5.000	46.00	16.00	22.11	0.348	0.481
6.000	40.00	25.00	21.17	0.625	0.529
7.000	38.00	21.00	22.99	0.553	0.605
8.000	42.00	27.00	27.88	0.643	0.664
9.000	42.00	28.00	30.22	0.667	0.719
10.00	43.00	38.00	35.35	0.884	0.822

Supplementary Figure S3. Multicollinearity Assessment

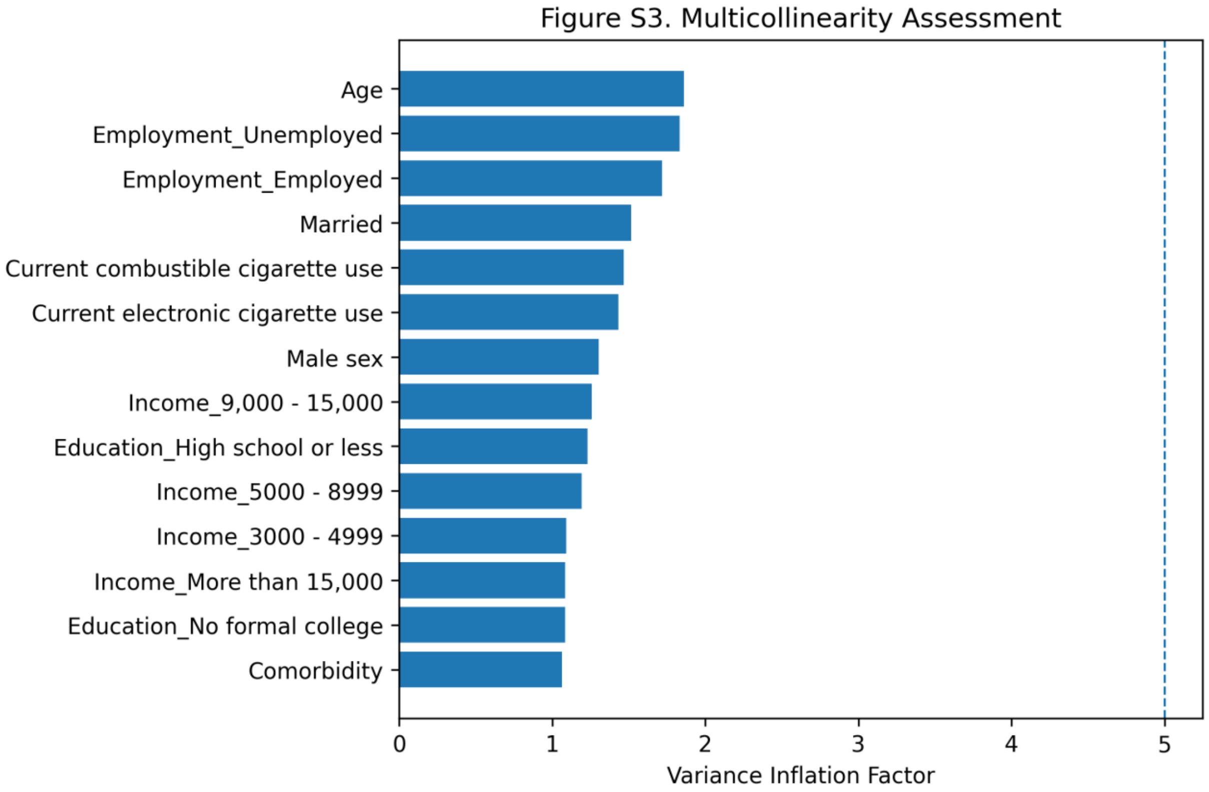

Variance inflation factors were examined for all predictors. The maximum VIF was 1.86, indicating no severe multicollinearity.

Supplementary Table S4. Variance Inflation Factors

Predictor	VIF
Age	1.860
Employment_Unemployed	1.833
Employment_Employed	1.718
Married	1.515
Current combustible cigarette use	1.468
Current electronic cigarette use	1.433
Male sex	1.303
Income_9,000 - 15,000	1.260
Education_High school or less	1.232
Income_5000 - 8999	1.193
Income_3000 - 4999	1.090
Income_More than 15,000	1.084
Education_No formal college	1.083
Comorbidity	1.062

Supplementary Figure S4. Influence and Residual Diagnostics

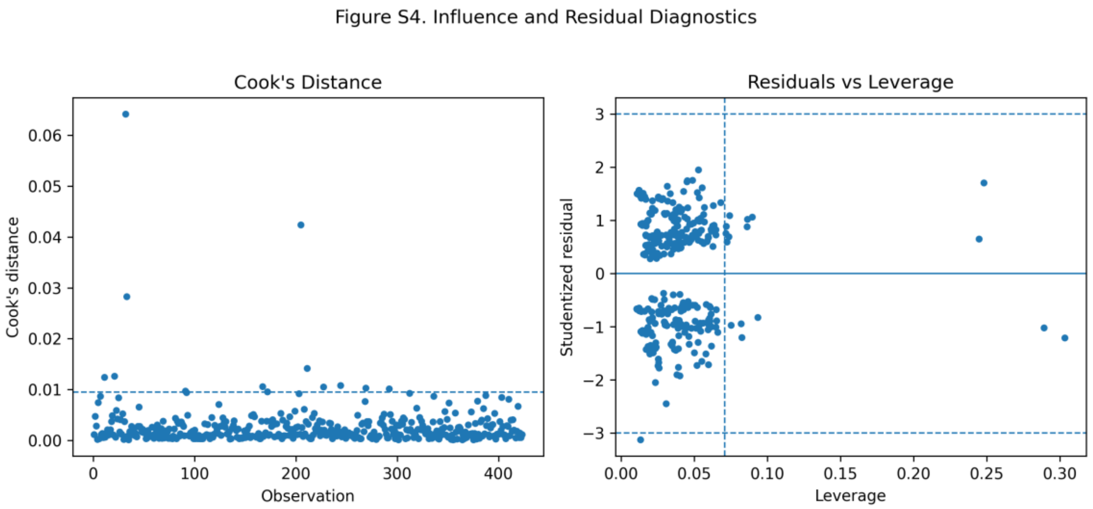

Influence diagnostics did not identify observations with undue impact on model estimates. Maximum Cook's distance was 0.064; the common 4/n threshold was 0.009. Maximum leverage was 0.303.

Supplementary Figure S5. Predictor Correlation Matrix

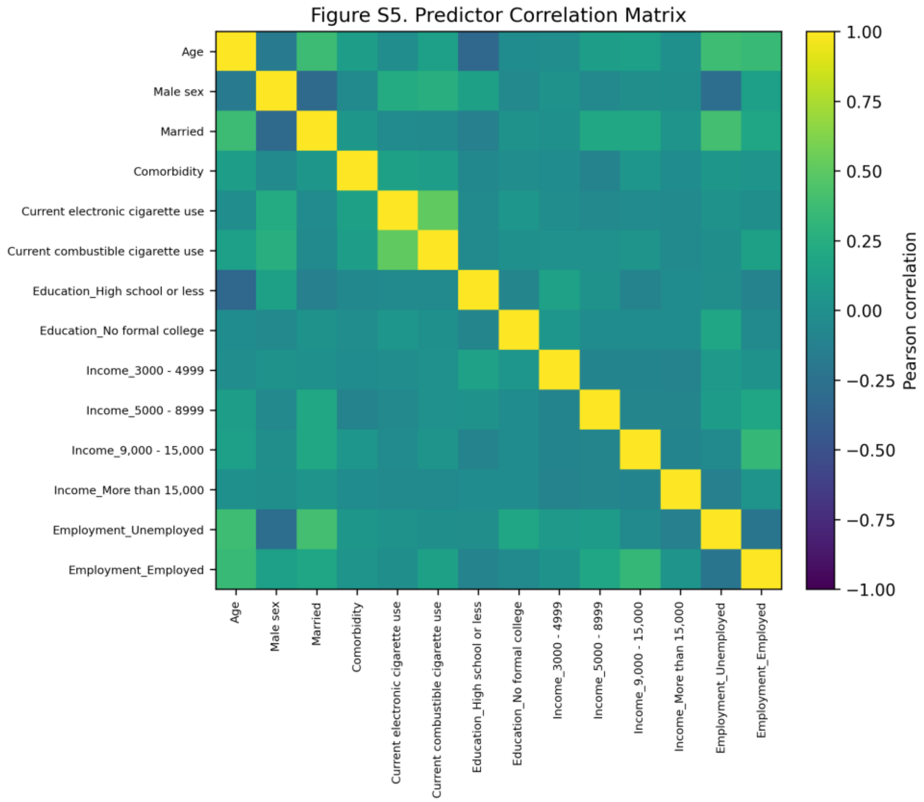

The predictor correlation matrix did not show a pattern suggestive of severe pairwise collinearity among variables included in the adjusted model.
